# Neurotransmitters Affect Larval Development by Regulating the Activity of Prothoracicotropic Hormone-Releasing Neurons in *Drosophila melanogaster*

**DOI:** 10.3389/fnins.2021.653858

**Published:** 2021-12-17

**Authors:** Shun Hao, Julia Yvonne Gestrich, Xin Zhang, Mengbo Xu, Xinwei Wang, Li Liu, Hongying Wei

**Affiliations:** ^1^State Key Laboratory of Brain and Cognitive Science, CAS Center for Excellence in Biomacromolecules, Institute of Biophysics, Chinese Academy of Sciences, Beijing, China; ^2^College of Life Sciences, University of the Chinese Academy of Sciences, Beijing, China; ^3^CAS Key Laboratory of Mental Health, Institute of Psychology, Chinese Academy of Sciences, Beijing, China

**Keywords:** PTTH neurons, spontaneous activity, acetylcholine, octopamine, *Drosophila* larvae, pupariation

## Abstract

Ecdysone, an essential insect steroid hormone, promotes larval metamorphosis by coordinating growth and maturation. In *Drosophila melanogaster*, prothoracicotropic hormone (PTTH)-releasing neurons are considered to be the primary promoting factor in ecdysone biosynthesis. Recently, studies have reported that the regulatory mechanisms of PTTH release in *Drosophila* larvae are controlled by different neuropeptides, including allatostatin A and corazonin. However, it remains unclear whether neurotransmitters provide input to PTTH neurons and control the metamorphosis in *Drosophila* larvae. Here, we report that the neurotransmitters acetylcholine (ACh) affect larval development by modulating the activity of PTTH neurons. By downregulating the expression of different subunits of nicotinic ACh receptors in PTTH neurons, pupal volume was significantly increased, whereas pupariation timing was relatively unchanged. We also identified that PTTH neurons were excited by ACh application *ex vivo* in a dose-dependent manner *via* ionotropic nicotinic ACh receptors. Moreover, in our Ca^2+^ imaging experiments, relatively low doses of OA caused increased Ca^2+^ levels in PTTH neurons, whereas higher doses led to decreased Ca^2+^ levels. We also demonstrated that a low dose of OA was conveyed through OA β-type receptors. Additionally, our electrophysiological experiments revealed that PTTH neurons produced spontaneous activity *in vivo*, which provides the possibility of the bidirectional regulation, coming from neurons upstream of PTTH cells in *Drosophila* larvae. In summary, our findings indicate that several different neurotransmitters are involved in the regulation of larval metamorphosis by altering the activity of PTTH neurons in *Drosophila*.

## Introduction

Animal development is a precise and complex process that allows each animal to choose the optimal time and appropriate environment for completing the transition from juvenile to adult. Adaptive mechanisms have evolved to generate optimized outputs in many species, and to maximize survival in varying natural circumstances, through neuroendocrine signaling (Gotthard and Nylin, 1995; Denver, 1997; Lessells, 2008). In insects, this transition is mainly controlled by the combined endocrine effects of several pivotal hormones and neuropeptides, including ecdysone, which is the master regulator of metamorphosis. Molting and metamorphosis correspond to peaks of ecdysone production, which are induced by prothoracicotropic hormone (PTTH). PTTH, a brain-derived neuropeptide hormone, is the primary promoter of ecdysteroidogenic activity in the prothoracic gland (PG). It is also required for the normal initiation of metamorphosis in several insect species, including the tobacco hornworm *Manduca sexta*, the silkworm *Bombyx mori*, the Colorado potato beetle *Leptinotarsa decemlineata*, and the fruit fly *Drosophila melanogaster* (Kawakami et al., 1990; Mizoguchi et al., 1990; Kataoka et al., 1991; Tomioka et al., 1995; McBrayer et al., 2007; Yamanaka et al., 2013a,b; Meng et al., 2019). The endocrine physiology of vertebrates and insects shows intriguing parallels and homologies. In both vertebrates and insects, steroid hormone biosynthesis is controlled by peptide hormones: for example, ecdysone synthesis is controlled by PTTH in insects, and glucocorticoids of the adrenal glands are regulated by adrenocorticotropic hormone (ACTH), a functional ortholog of PTTH, in vertebrates. Both of them show a pulsatile mode of secretion and promote the release of cortisol and ecdysone, respectively, which ensures the normal development and maturation of individuals at the proper time (Lightman and Conway-Campbell, 2010; McBrayer et al., 2007; Rewitz et al., 2009a; Stavreva et al., 2009; Nässel and Winther, 2010; Tennessen and Thummel, 2011; Di Cara and King-Jones, 2016).

Many previous studies of lepidopterans support the classical scheme, that the release of PTTH in the brain is the key that leads to peak levels of ecdysteroid biosynthesis for developmental transitions. According to this hypothesis, PTTH neurons evaluate and integrate various environmental and developmental inputs to determine when to transition to the next developmental stage (Truman and Riddiford, 1974; Gilbert et al., 1981; McBrayer et al., 2007). However, recent evidence shows that the PG plays a central role in developmental coordination, directly receiving multiple inputs from different upstream signaling pathways (Huang et al., 2008; Rewitz et al., 2009a,b; Yamanaka et al., 2013a,b; Koyama et al., 2020). There are many elements, such as nutritional cues, that represent the general metabolic status of each larva and act directly on ecdysone producing-cells in the PG to influence the timing of metamorphosis (Danielsen et al., 2013; Smith et al., 2014; Moeller et al., 2017). Some of these regulators likely suppress the ecdysteroidogenic activity of the PG until certain physiological conditions are met in a disinhibited mechanism. According to this concept, PTTH is still a major promoting factor of ecdysteroidogenesis (Isaacson and Scanziani, 2011; Yamanaka et al., 2013a,b; Letzkus et al., 2015; Jovanic et al., 2016). Furthermore, the peak release of ecdysone always follows pulses of PTTH release, just before each larval ecdysis and pupariation (Mizoguchi et al., 2002; McBrayer et al., 2007). A range of environmental and internal factors (such as photoperiod, temperature, and the nutritional state of each animal) have been reported to affect PTTH release, which concurrently affects larval development or behavioral characteristics (Gilbert et al., 1981; Schiesari et al., 2011; Di Cara and King-Jones, 2013; Liu et al., 2015; Cardinal-Aucoin and Steel, 2016; Selcho et al., 2017; Shimell et al., 2018). Additionally, PTTH neurons in the brain may receive many diverse regulatory inputs from different upstream neurons. There may also be other complex regulatory mechanisms at the level of PTTH neurons, which form an upstream control center that regulates the physiological activity of the PG.

The fruit fly *D. melanogaster* is a well-suited insect model for studying the mechanisms of PTTH production and release in response to developmental and environmental inputs. Two pairs of PTTH-releasing neurons are located in each hemisphere of the central brain in *Drosophila* larvae. They project and secrete PTTH neuropeptide into the PG. PTTH activates a canonical mitogen-activated protein kinase pathway *via* its receptor, torso, to promote ecdysone production for regulating larval maturation. Either the *ptth* gene mutant or the ablation of PTTH neurons delays the time to pupariation by 4 or 5 days and increases the body size of pupae in *Drosophila* larvae (McBrayer et al., 2007; Grillo et al., 2012; Smith and Rybczynski, 2012; Johnson et al., 2013). A recent study demonstrated that PTTH signaling is also a central component that contributes to the coordination between environmental cues and developmental status to ensure individual fitness and survival. Environmental inputs, such as nutrition and population density, can change the larval growth rate and pupal volume by affecting PTTH release to ensure optimal survival (Shimell et al., 2018). Recently, several upstream neural pathways of PTTH neurons have been discovered, demonstrating that a synthetic regulatory mechanism exists at the level of PTTH neurons. It has been reported that both allatostatin A (AstA)-producing neurons and corazonin (Crz) neurons form synaptic connections with PTTH neurons in the larval brain and increase PTTH release by activating PTTH neurons to promote larva-to-pupa transition. AstA neurons control PTTH secretion through the AstA receptor 1 (AstAR1), which is expressed on PTTH neurons, to start metamorphosis. Silencing AstA expression in the larval brain delays the onset of maturation and extends the growth period. Hence, AstA/AstAR1 signaling contributes to the balance of development and maturation (Deveci et al., 2019). Crz neurons are another kind of upstream neuron, and function only during the mid-third instar larval (L3) stage. The inhibition of Crz neuronal activity increases pupal size by enhancing the growth rate and delaying ecdysteroid elevation during the mid-L3 stage, but has almost no effect on pupariation timing (Imura et al., 2020). In addition, pigment-dispersing factor (PDF)-producing neurons are considered to be upstream neurons that influence larval phototaxis and pupal eclosion in *Drosophila*. A previous GFP reconstitution across synaptic partners (GRASP) and immunohistochemical study demonstrated that the axonal projection regions of PDF neurons remain fairly close to PTTH neurons in both larval and adult brains. Furthermore, rhythmic changes in PTTH transcript levels indicate that pacemaker neurons could temporally control PTTH production. Therefore, PTTH neurons receive circadian information from PDF neurons *via* the inhibitory short neuropeptide F precursor (sNPF) and then transmit time information to the PG *via* PTTH. The synchronization of peripheral clock in the PG with central brain clock could be essential for larval normal development (McBrayer et al., 2007; Gong et al., 2010; Di Cara and King-Jones, 2016; Selcho et al., 2017). The results of these previous studies indicate that all of the neurochemical molecules that directly act on PTTH neurons belong to neuropeptides, including AstA, Crz, and sNPF. However, it remains unclear whether classical neurotransmitters that are widespread in the central nervous system are involved in regulating the activity of PTTH neurons in *Drosophila* larvae. There is evidence that various neurotransmitters affect both steroid hormone release and the time of metamorphosis in other insects, such as lepidopterans, *B. mori*, *M. sexta*, and cockroaches (Lester and Gilbert, 1987; Shirai et al., 1994; Aizono et al., 1997; Noguchi and Hayakawa, 1997; Richter et al., 2000; Moulos et al., 2016). For example, cholinergic transmission might directly regulate PTTH release from neurosecretory cells in *B. mori* (Aizono et al., 1997). However, whether neurotransmitters participate in the regulatory network of PTTH release, and how this might affect larval maturation in *Drosophila*, remains elusive. Understanding the mechanisms of PTTH production and release will be key to deciphering the process of metamorphosis in insects.

The current study first analyzed the electrophysiological activity of PTTH-releasing neurons using patch-clamp techniques in *Drosophila* larvae. In these experiments, we measured electrophysiological aspects that are thought to influence and represent PTTH production and release. We revealed that PTTH neurons produced spontaneous action potentials (APs) at a low firing frequency *in vivo*. This characteristic of PTTH neurons indicates a physiological status for the benefits of developmental adjustment in a bidirectional manner. Next, we tested the Ca^2+^ response of PTTH neurons to two different neurotransmitters, acetylcholine (ACh) and octopamine (OA), which have previously been reported to be associated with PTTH release (Aizono et al., 1997; Ohhara et al., 2015; Imura et al., 2020). The Ca^2+^ imaging results revealed that ACh increased neural activity by acting on PTTH neurons directly, in a dose-dependent manner. Additionally, the pharmacological experiments demonstrated that ACh played an active role in regulating the activity of PTTH neurons *via* ionotropic nicotinic ACh receptors. Next, we found that downregulating the expression of nAChR, but not muscarinic ACh receptors (mAChR), in PTTH neurons led to increased pupal size without affecting pupariation timing in *Drosophila* larvae. This result was confirmed by Ca^2+^ imaging experiments in which we specifically downregulated the expression of different AChRs on PTTH cells using RNA interference (RNAi) techniques. Interestingly, the other neurotransmitter that we tested, OA, had different regulatory effects on PTTH neurons depending on its concentration. Lower doses of OA resulted in increased Ca^2+^ levels, while higher doses of OA resulted in decreased Ca^2+^ levels. Furthermore, using Ca^2+^- imaging and fluorescence resonance energy transfer (FRET)-based cAMP imaging on *ex vivo* larval brains, we identified that OA was conveyed through an OA beta (Octβ)-type receptor expressed on PTTH neurons. We propose that the inputs for these neurotransmitters might come from environmental information, such as the photoperiod, temperature, or some other factors. Taken together, our results indicate that neurotransmitters play a critical role in regulating the activity of PTTH neurons to coordinate larval growth and maturation and ensure normal individual development.

## Materials and Methods

### *Drosophila* Strains

As described by Guo et al. (1996), third instar flies were reared on a standard medium of corn meal and molasses at 60% relative humidity in a 12 h: 12 h light/dark cycle. For whole cell patch-clamp recordings, larvae of a PTTH-Gal4 (obtained from Christian Wegener’s lab, University of Würzburg) or 103604 (NP0394-Gal4; Drosophila Genetic Resource Center (DGRC) Kyoto) line specific for PTTH-expressing neurons (Yamanaka et al., 2013a,b) were crossed with flies of the UAS-mCD8:GFP strain (BL5137 from Bloomington *Drosophila* Stock Centers, BDSC). Crosses of the lines PTTH-Gal4 with flies of the UAS-GCaMP6m strain and PTTH-GAL4 with flies of the UAS-Epac-camps (BL25408) strain were used for Ca^2+^ imaging and Epac-camps FRET imaging experiments in *ex vivo* preparations. In the Calcium imaging experiments combined with AChR RNAi, we crossed the homozygous flies (UAS-GCaMP6m; PTTH-Gal4) expressing GCaMP6m in PTTH neurons with various AChR RNAi fly stocks. The flies, PTTH-Gal4 line crossed with different neurotransmitter receptors RNAi stocks, were analyzed in the developmental experiments. The fly stocks were used in RNAi experiments as follows: The stocks from Tsinghua *Drosophila* stock cener: UAS-*nAChR*α*1*-RNAi (THU3068), UAS-*nAChR*α*3*-RNAi (THU2756). The stocks from Bloomington *Drosophila* Stock Center: UAS-*mAChR-B*-RNAi (67775), UAS-*mAChR-C*-RNAi (61306).

### Electrophysiology

Whole cell patch-clamp recordings of PTTH neurons in the larval brain of *Drosophila* were exercised *in vivo*, using the following protocol. The ventral part of a third instar larva was fixed with dental glue (3 M, Vetbond) on the bottom of a recording chamber. Afterward, larval extracellular Ringer’s solution (101 mM NaCl, 3 mM KCl, 4 mM MgCl_2_, 1 mM CaCl_2_, 5 mM glucose, 20.7 mM NaHCO_3_, 1.25 mM NaH_2_PO_4_, pH 7.2) was supplied, and the perineural organ was gently removed with tweezers. All electrophysiological recordings were performed using a Nikon upright microscope (NI-FLT6) equipped with a mercury lamp (OSRAM) and a GFP filter (BP 450–480). The PTTH neurons were identified by their fluorescence and morphology under a 40x objective, and glass capillaries (1–2 MΩ, pulled from the glass of the type GCF150-7.5, Harvard Apparatus, United States) were used to gently remove upper neurons and superficial glia *via* applying negative pressure to expose PTTH neurons without disrupting them. During all of the recordings, the recording chamber was continuously perfused with larval extracellular Ringer solution and bubbled with 95% O_2_ and 5% CO_2_ (2 mL/min). Whole patch-clamp recordings were carried out with borosilicate glass capillaries (B15024F, Vitalsense Scientific Instruments Co., Ltd.) of about 8∼10 MΩ using a P97 micropipette puller (Sutter Instruments, Novato, United States) and filled with internal solution (102 mM K-gluconate, 0.085 mM CaCl_2_, 0.94 mM EGTA,8.5 mM HEPES, 1.7 mM MgCl_2_, 17 mM NaCl, adjusted to 235 mOsm, pH 7.2). Minimal suction followed by break in into whole cell configuration using the voltage clamp mode with a holding voltage of −80 mV allowed to achieve gigaseals. Afterward, the current-clamp mode was chosen to record the spontaneous firing of the cells. An Axon-700B MultiClamp amplifier acquired and digitized signals at 25 kHz and a filter was applied at 2 kHz with 4-pole Bessel filter using a Digidata 1550 (Axon Instruments, California, United States) converter. Traces of action potentials were analyzed with Clampex 9.0 software (Molecular Devices). The maximum action potential frequencies were calculated by Spikes2.0 software.

### Ca^2+^ Imaging and Epac-camps FRET Experiments

The brain tissues of early third instar larvae were dissected in *Drosophila* larval extracellular Ringer’s solution. Subsequently, each dissected brain was attached to the bottom of the perfusion chamber, with the anterior side up. The bottom of the chamber was coated with polylysine (0.2 mg/ml) in advance. Larval brains were continuously superfused with Ringer solution or stimulus presentation during the entire imaging experiment. Baseline fluorescences were acquired for 2 min before applying drugs or neurotransmitters. Brains were superfused until the fluorescences were restored to the basal level after stimulus treatments. To monitor Ca^2+^- and cAMP-induced fluorescence changes of GCaMP6m and Epac-camps expressing cells, we used a ZEISS LSM 880 confocal laser scanning microscope and a 20x/1.0NA W Plan-Apochromat DIC water-immersion objective (Carl Zeiss MicroImaging GmbH). All Ca^2+^ imaging experiments were scanned with a 488-nm laser. In each larval preparation, the calcium level of one PTTH neuron was analyzed. The cell bodies were defined for the region of interest (ROI), in which the GCaMP6m fluorescence intensities were measured. For individual PTTH neurons, calcium signal per frame was calculated as an average of all pixels within its ROI. This calculation was repeated for each frame in the time series to generate a single-neuron time course. The fluorescent signal was expressed as the percent change relative to the prestimulus baseline F_0_. The baseline fluorescence F_0_ was defined as average fluorescence during the 1-min period right before the beginning of stimulus application. The fluorescent signal for each PTTH neuron was calculated as the change of fluorescence intensity (F–F_0_ = ΔF) divided by the baseline fluorescence (ΔF/F_0_).

To characterize the calcium responses to ACh, we constructed dose-response curves based upon the normalized amplitude of the response. The response amplitude was defined as the absolute value from baseline to the peak of the stimulation-induced Ca^2+^ levels. The amplitude increased with the increasing concentration of neurotransmitters. Each recorded PTTH neuron reached a similar saturated response amplitude with 500 μM neurotransmitter application. Desensitization became apparent at higher concentrations (1 mM). Therefore, response amplitude to 500 μM ACh was used as reference (=100%) to calculate the dose-response relationship. All response amplitudes were normalized to this value. The normalized values were fitted to a 4-parameter dose-response curve with variable slope and plotted on logarithmic scales using SigmaPlot. Half-maximal effective concentration (EC50) was calculated from dose-response curves.

Epac-camps FRET imaging was performed by scanning frames with a 458-nm laser and a frequency of 0.83 Hz. The CFP and YFP emission (460–520 nm/520–580 nm) were separated by a Carl Zeiss confocal splitter. Background subtraction was performed for CFP and YFP intensities. The CFP/YFP ratio was calculated for each time point.

### Drug Application

To analyze the response of PTTH neurons to potential neurotransmitters, different doses of neurotransmitters ACh (100 nM–1 mM), OA (10 μM–1 mM) were applied for 60 s perfusion. In corresponding vehicle control groups, the application of neurotransmitters was replaced by Ringer’s solution. Next, we used the sodium channel blocker tetrodotoxin (TTX) (Taizhoukangte, 1 μM) to block synaptic transmission from other neurons. Brain tissues were incubated with TTX for 5 min at least. Afterward, TTX (1 μM) with the respective neurotransmitter was perfused simultaneously into the brain sample. We applied different specific AChR antagonists to further identify their receptor types. The brains were incubated with 100 μM scopolamine (Scop, the blocker of metabotropic acetylcholine receptors) (Gorczyca et al., 1991) or 100 μM mecamylamine (MCA, the blocker of nicotinic acetylcholine receptors) (Huang et al., 2010) before being treated with ACh (1 mM). To analyze the OA receptors, the neurons were incubated with 300 μM Ca^2+^ channel antagonist cadmium chloride (CdCl_2_) for 5 min before being treated with OA (50 μM). According to the dose-response dependent curve, we perfused a low concentration of OA (100 μM) to the brain and recorded the change of cAMP levels in PTTH neurons with Ringer solution as a control group. All drugs were purchased from Sigma Aldrich.

### Reverse Transcription Polymerase Chain Reaction

To confirm the RNAi efficacy, RT-PCR was performed following the protocol described in a previous study (Li et al., 2014). The RNAi lines were crossed with the *elav*-Gal4 line (BL458 from BDSC). *Actin* was used as the internal control. The expression level was calculated using AlphaEaseFC. The primers used in RT-PCR were as follows: primer of *nAChR*α*1* up: 5′-G CTGGCTGGTGGAATCTA-3′; primer of *nAChR*α*1* low: 5′-AG CACTGCCGATGACACT-3′; primer of *nAChR*α*3* up: 5′-GC GGGCGTCTGTGATATT-3′; primer of *nAChR*α*3* low: 5′-TGTG CTCTCCTCTTCCTTG-3′; primer of *mAChR-B* up: 5′-ATCTC TGGCTTTCCGTCG-3′; primer of *mAChR-B* low: 5′-TGAA GAAGAGCAGAGCGG-3′; primer of *mAChR-C* up: 5′-GAA GAGCGTCCAGATTGT-3′; primer of *mAChR-C* low: 5′-CGG ATTGAGAGCAGAGTTG-3′; primer of *actin* up: 5′-CAGG CGGTGCTTTCTCTCTA-3′; primer of *actin* low: 5′-AGCTGT AACCGCGCTCAGTA-3′.

### Developmental Experiments

All developmental experiments were performed at 25°C in 12 h light/dark cycle conditions. To maximize the fecundity, 150 female flies were crossed with 50 male flies for 3 days. Before actual collections, adults were allowed to lay for 1 h to remove held eggs. The flies were transferred to standard cornmeal food plus yeast paste for 1.5− to 2-h egg laying. The collected embryos laid on cornmeal food were incubated at 25°C for 110 h.

### Developmental Timing Analysis

The times of pupariation were scored every 3 h from 8:00 to 23:00 at 110 h after egg laying. Data were compiled and ordered by progressive pupariation time and cumulative percentage pupariation in Microsoft Excel and subsequently analyzed in GraphPad Prism.

### Measurements of Pupal Volume

Pupae were aligned in a petri dish with the dorsal upward and photographed under a microscope after most of the pupae turned black, at which point the gender could be distinguished according to the eye color or by dissecting the pupae. In each group, the volumes of ten male and female pupae were measured with ImageJ. The pupal length was determined along the medial line between anterior and posterior, not including the anterior and posterior spiracles. Pupal width was measured along the axial line. Pupal volume was calculated by using the formula for a prolate spheroid: (π/6) W^2^ × L, where W was pupal width and L was the pupal length (Shimell et al., 2018; Deveci et al., 2019).

### Statistical Analyses

Data were given as means ± standard errors of the mean. Statistical analysis was performed with GraphPad Prism 6.0. The normality of all data groups was examined with the D’Agostino and Pearson omnibus normality test. For statistical analysis of RT-PCR data (Supplementary Figure 1), *t*-test was used to compare the mRNA expression in RNAi knockdown experimental groups with the corresponding control groups. For statistical analysis of developmental data (Figures 3A1,A2), one-way ANOVA was applied to analyze the mean difference among three normal distributed groups of data. Tukey test method was performed for the correction of multiple comparisons. Kruskal–Wallis test was applied to analyze the mean difference among three groups of data which include at least one non-normal distributed group. In calcium/cAMP imaging experiments, n indicated the number of tested brains. To identify the AChR (Figure 2D), the amplitudes of Ca^2+^ transients in the same cells before and after blocker treatment were compared by use of the paired *t*-test. For statistical analysis of calcium imaging data in Figure 4C, an unpaired *t* test was used to compare the difference between two normally distributed data groups. Non-normal distributed data groups were statistically analyzed by Wilcoxon signed rank test. The significance levels of the statistical tests were presented as *p < 0.05, ^**^p < 0.01, ^***^ *p* < 0.001; ns, not significant *p* > 0.05.

**FIGURE 1:**
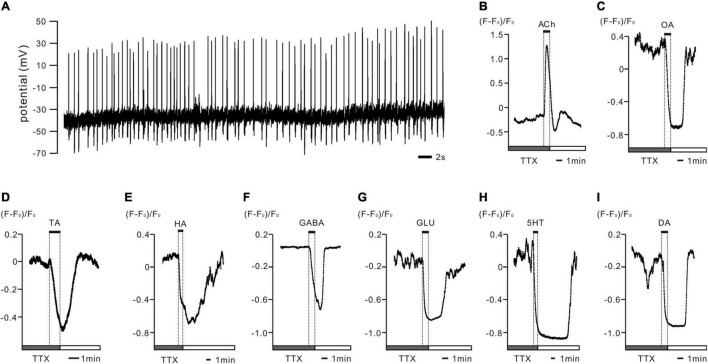
*In vivo* recorded PTTH neurons displayed spontaneous activity patterns and were shown to be regulated by various neurotransmitters in *ex vivo* preparations of *Drosophila* larval brains. **(A)** The representative whole cell patch clamp recording trace of PTTH cell *in vivo* shows spontaneously firing action potentials (APs) throughout the day. All PTTH neurons recorded presented a tonic firing pattern at a frequency of 1.26 ± 0.14 Hz (*n* = 15). **(B–I)** Representative delta*F*/*F* traces of Ca2+ responses in GCaMP6m-expressing PTTH neurons after tetrodotoxin (TTX) disrupted AP-dependent synaptic transmission. Application of 1 mM acetylcholine (ACh) to the bath solution resulted in increased intracellular Ca^2+^ concentrations (*n*_ACh_ = 10), whereas octopamine (OA), tyramine (TA), histamine (HA), γ-aminobutyric acid (GABA); glutamate (GLU), serotonin (5-HT) and dopamine (DA) decreased intracellular Ca^2+^ levels in all of the imaged PTTH cells (*n*_OA_ = 5; *n*_TA_ = 4; *n*_HA_ = 8; *n*_GABA_ = 8; *n*_GLU_ = 7; *n*_5–HT_ = 6; *n*_DA_ = 7). Gray bars display 5 min stimulus applications of 1 μM TTX. Solid bars indicate 1 min duration of the respective neurotransmitter application.

**FIGURE 2:**
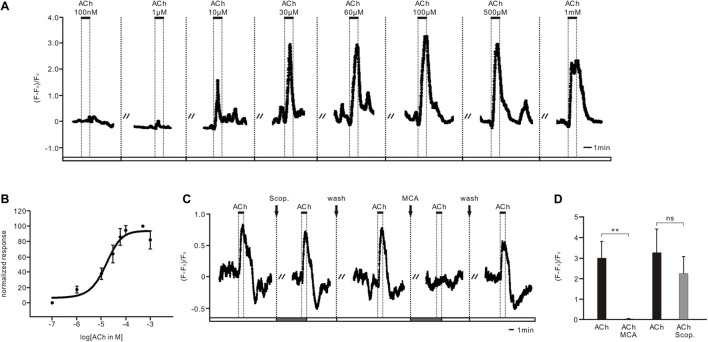
Acetylcholine (ACh) increased dose-dependently intracellular Ca^2+^ concentrations *via* a nicotinic but not a muscarinic ACh receptor in PTTH cells. **(A)** Ca^2+^ imaging from *ex vivo* preparations of GCaMP6m-expressing PTTH cells revealed dose-dependent reversible increases in intracellular Ca^2+^ levels after ACh application (100 nM–1 mM; *n* = 10). At higher concentrations of ACh, intracellular calcium levels of PTTH cells expressed fast desensitization. **(B)** Normalized ACh dose–response curve. EC_50_ = 22.65 μM. Data were plotted as mean ± SE. **(C)** ACh responses could not be blocked through pre-incubation (5 min) with the muscarinic ACh receptor antagonist scopolamine (Scop, 100 μM). Instead, pre-incubation (5 min) with the nicotinic ACh receptor antagonist mecamylamine (MCA, 100 μM) blocked all ACh responses (*n* = 10). Dashed lines display separated consecutive stimulus applications of 5 min washes or antagonist applications. Solid bars indicate the duration of 1 min ACh application. **(D)** Increased calcium level in PTTH neurons in response to ACh was significantly blocked after MCA treatment (*n* = 8), but not affected by Scop treatment (*n* = 4). **p < 0.01; ns, not significant *p* > 0.05.

**FIGURE 3:**
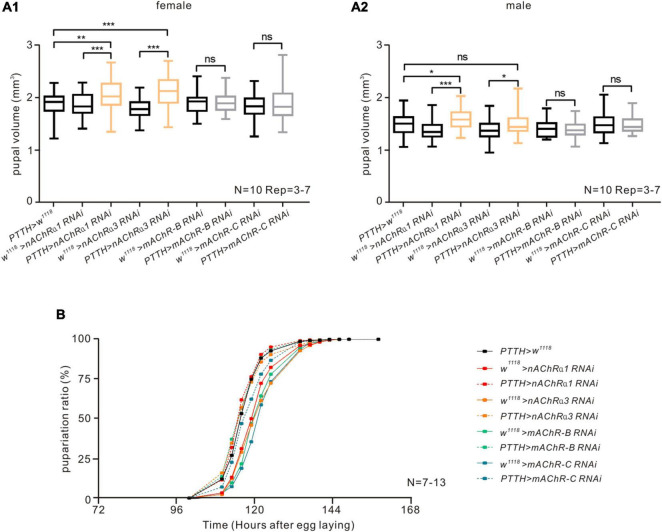
nAChRs on PTTH neurons have a negative effect on pupal volume but do not influence pupariation timing in *Drosophila*. **(A)** Downregulating the expression of nAChR α1 or α3 subunit in PTTH neurons caused an increase in pupal volume but no obvious changes in pupariation timing in both female **(A1)** and male **(A2)** flies. There was no change in pupal volume when downregulating the expression of mAChR-B/C subtypes in PTTH cells. The orange bars represent experimental groups with significant differences. We measured the volume of 10 female pupae and 10 male pupae in every experiment and repeated the experiment 3–7 times. *p < 0.05, **p < 0.01, *** *p* < 0.001; ns, not significant *p* > 0.05. **(B)** Downregulating the expression of different nAChR subunits (α1, α3) or mAChR subtypes (mAChR-B or mAChR-C) in PTTH neurons had no influence on pupariation timing. In the pupariation timing curves, different colors represent different receptor RNAi control groups (solid lines) and experimental groups (dashed lines). The experiment in all groups was repeated 7–13 times.

**FIGURE 4:**
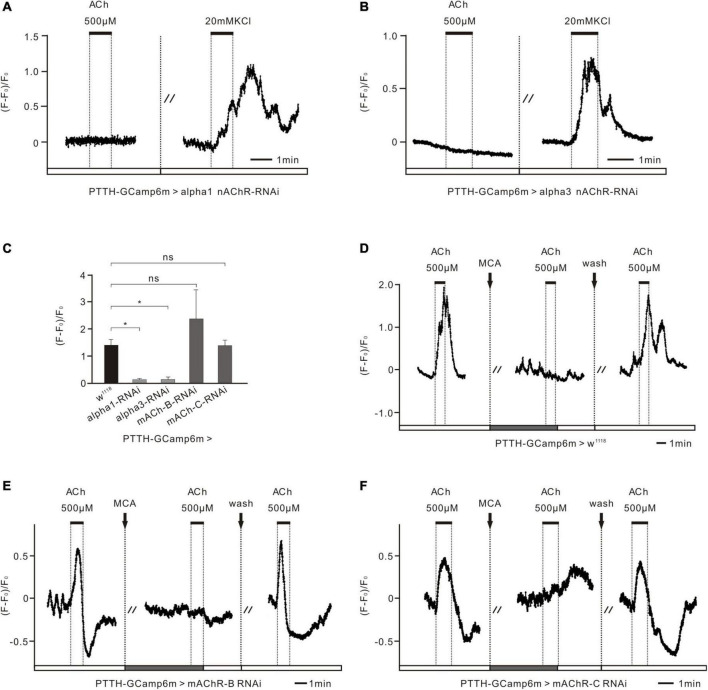
nAChR subunits control the excitatory response of PTTH neurons to ACh in *Drosophila* larvae. **(A,B)** Ca^2+^ response of two nAChR subunits in the RNAi experimental groups (co-expressing GCaMP6m and nAChRα1/3 RNAi in PTTH neurons) to 500 μM ACh and 20 mM KCl (*n* = 5). When downregulating the expression of nAChR α1 and α3 subunits specifically in PTTH neurons whose activity was demonstrated to be normal by the application of 20 mM KCl, no ACh response was detected. **(C)** Statistical analysis of the peak amplitude of Ca^2+^ response to 500 μM ACh in all groups (*n* = 4–5). The peak amplitude of the response to 500 μM ACh fell to almost zero after the expression of nAChRα1/3 was downregulated in PTTH neurons. *p < 0.05; ns, not significant *p* > 0.05. **(D)** Ca^2+^ response of the *w*^1118^ control group to ACh and the nAChR antagonist mecamylamine (ACh, 500 μM; MCA, 100 μM; *n* = 5). 100 μM MCA was able to completely block the increased response of PTTH neurons to 500 μM ACh. **(E,F)** Ca^2+^ response of the other AChR RNAi experimental groups (co-expressing GCaMP6m and mAChR-B/C RNAi in PTTH neurons) to 500 μM ACh and 100 μM MCA (*n* = 4–5). There were no significant differences between the control group and these experimental groups **(C)**.

## Results

### Spontaneous Activity Is Generated in Prothoracicotropic Hormone Neurons and Is Regulated by Various Neurotransmitters

To examine the intrinsic physiological properties of PTTH neurons in *Drosophila* larvae, we used electrophysiological recordings and Ca^2+^ imaging. *In vivo* whole-cell patch-clamp recordings revealed that GFP-labeled PTTH cells exhibited spontaneous activity and fired APs in a tonic pattern (Figure 1A, *n* = 15). The firing frequency was 1.26 ± 0.14 Hz. To study the possible regulatory mechanisms of neurotransmitter signaling, we applied ACh, OA, TA, HA, GABA, GLU, 5-HT, and DA to GCaMP6m-expressing PTTH cells in *ex vivo* preparations and examined the responses using Ca^2+^ imaging. To ensure that the neurotransmitters directly modulated neuronal activity, we disrupted AP-dependent synaptic transmission with TTX. After TTX incubation, the co-application of 1 μM TTX and 1 mM ACh increased Ca^2+^ levels in PTTH neurons (*n* = 10). In contrast, the simultaneous treatment with 1 μM TTX and 1 mM of other tested neurotransmitters (OA, TA, HA, GABA, GLU, 5-HT, and DA) decreased Ca^2+^ concentrations in PTTH neurons (Figures 1B–I, *n*_OA_ = 5; *n*_TA_ = 4; *n*_HA_ = 8; *n*_GABA_ = 8; *n*_GLU_ = 7; *n*_5–HT_ = 6; *n*_DA_ = 7).

### ACh Increases Intracellular Ca^2+^ Levels *via* nAChRs

ACh treatment increased the intracellular Ca^2+^ levels of PTTH neurons in a dose-dependent manner (Figure 2A, *n* = 10). To calculate the dose dependency of the ACh responses, we measured the amplitude from the control level to the maximum intracellular Ca^2+^ increase. The threshold concentration of the dose-dependent ACh effect varied between 100 nM and 1 μM. The magnitude of the peak response was dose-dependent between 1 μM and 1 mM (Figures 2A,B; EC_50_ = 22.65 μM). Desensitization of ACh-sensitive PTTH neurons was observed at higher concentrations (1 mM, Figures 2A,B). To determine whether ACh responses were mediated by ionotropic nAChRs or metabotropic mAChRs, the mAChR antagonist scopolamine (Scop) or the nAChR antagonist mecamylamine (MCA) was applied. Pre-incubation with Scop (100 μM) did not block ACh responses in any of the cells tested (*n* = 10). In contrast, MCA (100 μM) blocked the effects of ACh in all of the tested PTTH neurons (Figure 2C; *n* = 10). The statistic also demonstrated that the maximum ACh response almost got to zero after incubating 100 μM MCA, which was significantly lower than that response without MCA (Figure 2D, *n* = 8). However, there was no difference in maximum ACh response between before and after the incubation of 100 μM Scop (Figure 2D, *n* = 4). Thus, all ACh responses of PTTH cells were conveyed through nAChRs, and not through mAChRs.

### nAChRs in Prothoracicotropic Hormone Neurons Are Involved in Regulating Pupal Size but Not Pupariation Timing

The findings of our pharmacological experiments suggest that nAChRs, but not mAChRs, are expressed on PTTH neurons. To evaluate whether AChRs on PTTH neurons affect the larval developmental process by altering neural activity, we analyzed the two most common parameters in *Drosophila* metamorphosis—pupal volume and pupariation timing, which reflect the larval holistic developmental state—because PTTH neurons are known to promote larval maturation (Kawakami et al., 1990; Tomioka et al., 1995; McBrayer et al., 2007; Rewitz et al., 2009a,b). In the present study, we downregulated the expression of different kinds of AChR subunits in PTTH neurons by crossing a PTTH-Gal4 line with corresponding RNAi lines of receptor subunits. RT-PCR analysis showed that the mRNA expression of *nAChR*α*1*, *nAChR*α*3, mAChR-B, and mAChR-C* was significantly reduced in *Drosophila* brains with RNAi knockdown in comparison with their corresponding control groups (Supplementary Figure 1). When the expression of nAChR α1 and α3 subunits, which are homologs of vertebrate nAChR α2 subunits, were specifically decreased in PTTH neurons, pupal size was significantly increased compared with control groups in both male and female flies (Figures 3A1,A2). In contrast, the time curve of pupariation percentage was unaffected (Figure 3B). In addition to the nAChR subunits, we performed developmental experiments in metabotropic mAChR subtypes, including mAChR-B and mAChR-C. Larvae with decreased expression of these two mAChR subtypes on PTTH neurons had no significant changes in pupal size or pupariation timing (Figures 3A1,A2,B). Together, these results suggest that nAChRs in PTTH cells participate in the regulation of larval metamorphosis, and especially growth rate, but that mAChRs do not.

### nAChRs in Prothoracicotropic Hormone Neurons Negatively Act on Pupal Volume by Directly Regulating the Activity of Prothoracicotropic Hormone Neurons

To further demonstrate that ACh functions by directly binding to nAChRs in PTTH cells, we applied 500 μM ACh to PTTH cells that had RNAi-induced decreased expression of different AChR subunits or subtypes in Ca^2+^ imaging experiments. In these experiments, we used the PTTH-Gal4 driver lines combined with UAS-GCaMP6m and UAS-AChR-RNAi to detect the Ca^2+^ response of PTTH neurons to ACh. When the expression of nAChR α1 and α3 subunits in PTTH neurons was decreased, intracellular Ca^2+^ levels were not affected by the perfusion of 500 μM ACh. To test the viability of the tested brains, Ca^2+^ responses to Ringer’s solution containing 20 mM KCl were examined. This solution depolarized intact cell membranes and promoted a voltage-dependent Ca^2+^ influx. The Ca^2+^ levels increased markedly with perfusion of 20 mM KCl. This finding indicates that PTTH neurons without nAChR α1 or α3 subunits are unable to produce intact and functional nAChRs, which contributes to a silent response to ACh (Figures 4A,B). In contrast, PTTH neurons with decreased mAChR-B and mAChR-C still had elevated intracellular Ca^2+^ levels when 500 μM ACh was applied, as did the *w*^1118^ control group (Figures 4D–F). Moreover, the increased reaction induced by ACh was completely blocked by 100 μM of the nAChR antagonist MCA, similar to what was observed in the pharmacological experiments (Figure 2C). Statistical analysis revealed that the Ca^2+^ response to ACh application in the nAChR α1 and α3 RNAi groups was significantly lower than that in the *w*^1118^ control group. In contrast, there were no significant differences between the mAChR-B/C RNAi groups and the *w*^1118^ control group (Figure 4C). These results were consistent with our aforementioned developmental results (Figures 3A1,A2). Together, these data indicate that ACh regulates the activity of PTTH neurons through nAChRs, but not mAChRs, to affect pupal volume in *Drosophila*.

### OA Plays a Bidirectional Role in the Activity of Prothoracicotropic Hormone Neurons Depending on Its Concentration

**FIGURE 5:**
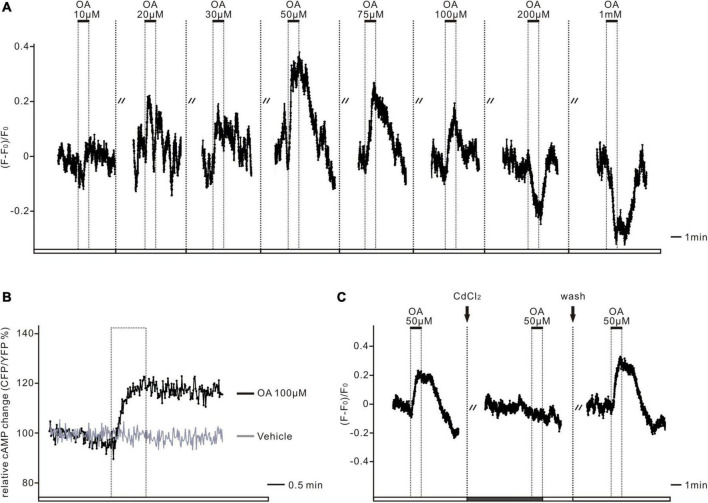
The dual-directional action of octopamine was mediated *via* OCTβ-type receptors. **(A)** The representative deltaF/F trace shows the Ca^2+^ response in one GCaMP6m-expressing PTTH neuron. Low doses of octopamine (OA) treatment (10–100 μM) initially led to increases, whereas higher doses (200 μM–1 mM) resulted in decreases of intracellular Ca^2+^ levels in all of the imaged PTTH cells (*n* = 7). Dashed lines display 5 min applications of washes. Solid bars indicate the duration (1 min) of the respective neurotransmitter application. **(B)** The Epac-camp inverse FRET trace for PTTH cells to bath application of 100 μM OA results in elevated cAMP levels in contrast to the vehicle control. The dashed box indicates the duration of the respective drug treatment. **(C)** 300 μM treatment of the voltage-dependent Ca^2+^ channel antagonist cadmiumchlorid (CdCl_2_) abolished OA (50 μM) induced Ca^2+^ increases in GCaMP6m-expressing PTTH cells (*n* = 6). Dashed lines display 1 min application of CdCl_2_ and 5 min application of washes. Solid bars indicate the duration of the respective drug application (1 min).

Depending on the OA concentrations that were added, the PTTH neurons had different responses. Higher concentrations of OA treatment (200 μM–1 mM) led to decreases in Ca^2+^. In contrast, the application of lower concentrations, in more common physiological ranges (10–100 μM), increased the intracellular Ca^2+^ levels of PTTH neurons (Figure 5A, *n* = 7). There are three classes of OA receptors in *D. melanogaster*: (1) OCTα-R, which is mainly associated with calcium release from the endoplasmic reticulum (ER) and an increase in intracellular Ca^2+^ level; (2) OCTβ-R, which is mainly coupled with an increase with intracellular cAMP level; (3) Octopaminergic/tyraminergic (OCT/TYR-R), which could be activated by both OCT and TYR, and associated with a decrease in cAMP level (Evans and Maqueira, 2005; Farooqui, 2012). To distinguish which receptor type was responsible for this Ca^2+^ increase, we next examined whether PTTH responses were mediated *via* the activation of adenylyl cyclase (AC), or by the release of Ca^2+^ from the ER. The cAMP imaging experiments demonstrated that cAMP levels increased after the bath application of 100 μM OA, whereas the control group (with the application of the bathing vehicle) had no changes (Figure 5B, *n* = 5). Adding the voltage-dependent Ca^2+^ channel antagonist CdCl_2_ (300 μM) prevented the OA-induced Ca^2+^ increases (Figure 5C, *n* = 6). This result indicates that OA-dependent AC/cAMP signaling may increase the opening probability of voltage-dependent Ca^2+^ channels and induce extracellular calcium influx, but not calcium release from ER. Thus, low concentrations of OA treatment are likely to activate PTTH neurons *via* Octβ-type receptors.

## Discussion

In the present study, we focused on neurotransmitter signaling to PTTH neurons, through which PTTH release can be procedurally regulated in *Drosophila* larvae to ensure normal development before the transition to adult flies. First, we used whole-cell patch-clamp recordings to characterize the physiological properties of PTTH neurons. We demonstrated for the first time that PTTH neurons were able to generate spontaneous APs in a tonic manner during the L3 stage. Next, we screened and classified the responses of PTTH neurons to eight kinds of typical neurotransmitters in the *Drosophila* brain *via* Ca^2+^- and EPAC-cAMP FRET imaging experiments. Similar to ACh, relatively low doses of OA were also able to increase the intracellular Ca^2+^ levels of PTTH neurons, whereas all of the other tested neurotransmitters (GLU, GABA, 5-HT, DA, TA, and HA) and higher concentrations of OA provided inhibitory inputs. We further examined how excitatory ACh receptors in PTTH neurons affected growth and maturation in *Drosophila* larvae. Knockdown of nAChR α1 and α3 in PTTH neurons significantly reduced the Ca^2+^ responses of PTTH neurons to ACh, and increased pupal volume.

### What Is the Functional Role of Excitatory ACh Signaling in *Drosophila* Larval Prothoracicotropic Hormone Neurons?

Our neurotransmitter screening revealed that ACh treatment led to dose-dependent Ca^2+^ increases *via* nAChRs in all tested PTTH neurons in the *Drosophila* larval brain. Hyperactivation/depolarization has been reported to play a critical role in PTTH release. For example, several studies have implied that cholinergic neurons can form synapses with prothoracicotropes and promote PTTH release (Lester and Gilbert, 1986, 1987; Agui, 1989; Shirai et al., 1994; Gilbert, 2009; Gong et al., 2010; Qin et al., 2019). Reports of *M. sexta* and *B. mori* brains have noted that high K^+^ concentrations together with Ca^2+^ cause depolarization of the treated neurons and leads to PTTH release (Carrow et al., 1981; Shirai et al., 1995). Furthermore, fifth instar larval *Manduca* brains have been demonstrated to release PTTH and increased ecdysteroid synthesis after ACh accumulation (Lester and Gilbert, 1986, 1987), suggesting a supportive role for the interaction between cholinergic neurons and PTTH. To date, evidence for PTTH release from cholinergic stimulation can also be drawn from *in vitro* studies. Cultured *Mamestra* brains release PTTH into the medium after the application of ACh and cholinergic agonists (Agui, 1989). Moreover, an *in vitro* study of the brains of *B. mori* showed similar results and highlighted the likely role of cholinergic transmission in regulating PTTH release from neurosecretory cells. However, the PTTH-producing neurosecretory cells of *B. mori* function *via* mAChRs, but not nAChRs (Aizono et al., 1997). This discrepancy with the present results may be caused by insect species variability. ACh/AChR have been reported to play an important role in circadian clock neurons (Keene et al., 2011; Keene and Sprecher, 2012), which are essential for steroid hormone production in *Drosophila* (Di Cara and King-Jones, 2016). Some anatomical and physiological studies regarding the role of ACh in the larval visual and circadian circuits implied clock neurons were supposed to be the most likely presynaptic input candidates for transmitting ACh to PTTH neurons. Cholinergic fifth lateral neurons in the *Drosophila* larval brain probably serve as potential upstream neurons that relay circadian input or light information to PTTH neurons *via* nAChRs (McBrayer et al., 2007; Johard et al., 2009; Keene and Sprecher, 2012).

Our data suggest that PTTH cells receive excitatory input from upstream cholinergic neurons *via* nAChRs to adjust the schedule of larval metamorphosis. On the one hand, the ubiquitous excitatory neurotransmitter ACh may relay a universal excitatory drive to PTTH neurons, responsible for the generally fast exchange of information flow between neurons. Because clock neurons receive light-induced input from photoreceptors on the retina and are described to form potential cholinergic synaptic contacts with PTTH neurons, we speculate that PTTH neurons might receive cholinergic inputs from clock cells, such as fifth lateral neurons (Johard et al., 2009; Keene et al., 2011; Yuan et al., 2011). This likely supports light avoidance behavior and promotes appropriate PTTH release, which is critical for larval survival before the transition to an adult fly. However, we also demonstrated that ACh/nAChR signaling is indeed involved in larval metamorphosis by directly exciting PTTH cells. When the expression of nAChR in PTTH cells was impaired, the disrupted balance between excitatory and inhibitory inputs resulted in an increased larval growth rate and pupal size but did not affect pupariation timing, which is very similar to the results of a previous study of the neuropeptide Crz in *Drosophila* larvae (Imura et al., 2020). Furthermore, our study also supports the idea that PTTH signaling controls ecdysteroid biosynthesis not only at peak levels but also at basal levels (Colombani et al., 2015; Moeller et al., 2017). Basal ecdysteroid negatively affects the larval growth rate without changing the pupariation timing.

### Bidirectional Actions of OA on the Activity of Prothoracicotropic Hormone Neurons

To date, little is known about the relationship between OA and PTTH neurons. However, there is some indication of their interaction in relation to larval metamorphosis. One study reported that Octβ3R in the PG plays an essential role in ecdysone biosynthesis. The knockdown of Octβ3R in PG leads to arrested metamorphosis because of a lack of ecdysone (Ohhara et al., 2015). Meanwhile, PTTH and insulin-like peptide signaling are also impaired after the downregulation of Octβ3R expression in the PG. Monoaminergic autocrine signaling in the PG is considered to regulate ecdysone biogenesis by integrating and coordinating PTTH and insulin-like peptide signaling, thus allowing metamorphosis to occur only when critical body weight is attained during larval development and nutrients are sufficiently abundant (Luo et al., 2012; Shimada-Niwa and Niwa, 2014). Additionally, another study demonstrated that a cluster of OA neurons in the subesophageal zone transmits nutrient signals to the PG *via* the Crz–PTTH neuronal axis to modulate ecdysteroid biosynthesis and negatively control systemic body growth during the mid-L3 in *Drosophila* larvae (Youn et al., 2018; Imura et al., 2020). Based on the results of these studies, we speculated that OA probably acts on PTTH cells, and affects cellular activity in a direct or indirect manner. Our Ca^2+^ imaging experiments demonstrated that OA led to either Ca^2+^ increases or decreases depending on the applied dose. Low-dose OA (≤100 μM) elevated the intracellular Ca^2+^ levels of PTTH cells, whereas high-dose OA (≥200 μM) had a clear inhibitory effect on PTTH cells. Next, we confirmed that receptor activation *via* low doses of OA, which were much closer to physiological concentrations *in vivo*, was associated with increased cAMP levels in PTTH cells (Figure 5B). We, therefore, hypothesized that low doses of OA might modulate the activity of PTTH cells *via* OctβRs, according to the classification and characteristics of OA receptors (Farooqui, 2012). This result is consistent with the expression pattern of Octβ1R, which has a wide distribution in the larval brain and especially high expression around the pars intercerebralis, the major region to which the dendrites of PTTH neurons project (El-Kholy et al., 2015). Thus, we consider that Octβ1R in PTTH cells has a positive effect on PTTH release. For the inhibitory effects at higher concentrations, we made preliminary speculation that high doses of OA might interfere with other signaling pathways, thus inducing some unphysiological changes; one possibility is that OA at high concentrations might bind and activate other analog receptors, such as tyramine receptors. The effect of bidirectional regulation of OA on PTTH neurons remains to be examined further.

In summary, the results of our study indicate that multiple neurotransmitters and their specific receptors contribute to maintaining the normal release of PTTH during larval development and metamorphosis by directly regulating the activity of PTTH neurons, which are also able to generate spontaneous APs in *Drosophila* larvae. Additionally, signal transmission to PTTH neurons is modulated by potentially opposing forces from diverse neurotransmitter inputs. Among these neurotransmitters, both ACh and low-dose OA activate PTTH neurons. The excitatory effect of ACh on PTTH neurons plays a restrictive role in the larval growth rate and pupal volume by activating PTTH neurons. In contrast, all of the other neurotransmitters that were investigated showed direct or indirect inhibitory effects on the activity of PTTH neurons. We thus speculate that the dynamic state of PTTH neurons is determined by direct and/or indirect interactions with various neurotransmitters, which determine the homeostasis of PTTH release at any given time. These input signals from different neurotransmitters probably come from respective upstream neural circuits, corresponding to continuous physiological and behavioral adjustments caused by external or internal conditional changes. Thus, PTTH cells likely maintain a low active state through a disinhibitory mechanism, and the larvae keep feeding and growing until all inhibitory conditions are removed. At this point, presynaptic neurons integrate all of the positive input signals to PTTH neurons and initiate a peak of PTTH release, which then promotes larval molting and metamorphosis. This mechanism coordinates signals from the external environment and internal homeostasis and ensures the normal process of larval development by adaptively altering the timing of steroid hormone biosynthesis. However, more evidence is needed to confirm this hypothesis.

## Data Availability Statement

The original contributions presented in the study are included in the article/Supplementary Material, further inquiries can be directed to the corresponding author/s.

## Author Contributions

SH, JG, LL, and HW designed the research. SH, JG, XZ, MX, XW, and HW performed the research. SH, JG, XZ, and HW analyzed the data. SH, JG, and HW wrote the manuscript. All authors contributed to the article and approved the submitted version.

## Conflict of Interest

The authors declare that the research was conducted in the absence of any commercial or financial relationships that could be construed as a potential conflict of interest.

## Publisher’s Note

All claims expressed in this article are solely those of the authors and do not necessarily represent those of their affiliated organizations, or those of the publisher, the editors and the reviewers. Any product that may be evaluated in this article, or claim that may be made by its manufacturer, is not guaranteed or endorsed by the publisher.

## Supplementary Material

Supplementary Figure 1 The mRNA expression levels of different *AChRs* in *Drosophila* brains were suppressed using the RNAi technique. The RT-PCR results shown are expressed as the ratio of AChR to actin. The mRNA expression of *nAChR*α*1* **(A)**, *nAChR*α*3* **(B)**, *mAChR-B* **(C)**, *and mAChR-C* **(D)** was significantly reduced in RNAi knockdown lines compared with compared with their corresponding control groups (**p* < 0.05, ^**^*p* < 0.01, and ^***^*p* < 0.001, *t*-test).

## Abbreviations

AC: adenylyl cyclase
ACh: acetylcholine
ACTH: adrenocorticotropic hormone
APs: action potentials
AstA: allatostatin A
CdCl_2_: cadmium chloride
Crz: corazonin
DA: dopamine
FRET: fluorescence resonance energy transfer
GRASP: GFP reconstitution across synaptic partners
GABA: γ-aminobutyric acid
GLU: glutamate
HA: histamine
MAPK: mitogen activated protein kinase
MCA: mecamylamine
OA: octopamine
PDF: pigment dispersing factor
PG: prothoracic gland
PTTH: prothoracicotropic hormone
PTX: picrotoxin
Scop: scopolamine
sNPF: short neuropeptide F precursor
TA: tyramine
TTX: tetrodotoxin
5-HT: (5-hydroxytryptamine) serotonin.
