# Supplementary figures and images for: Neurotransmitters Affect Larval Development by Regulating the Activity of Prothoracicotropic Hormone-Releasing Neurons in Drosophila melanogaster
Source: Front Neurosci. 2021 Dec 17;15:653858. doi: 10.3389/fnins.2021.653858 (PMC8718639; doi:10.3389/fnins.2021.653858)

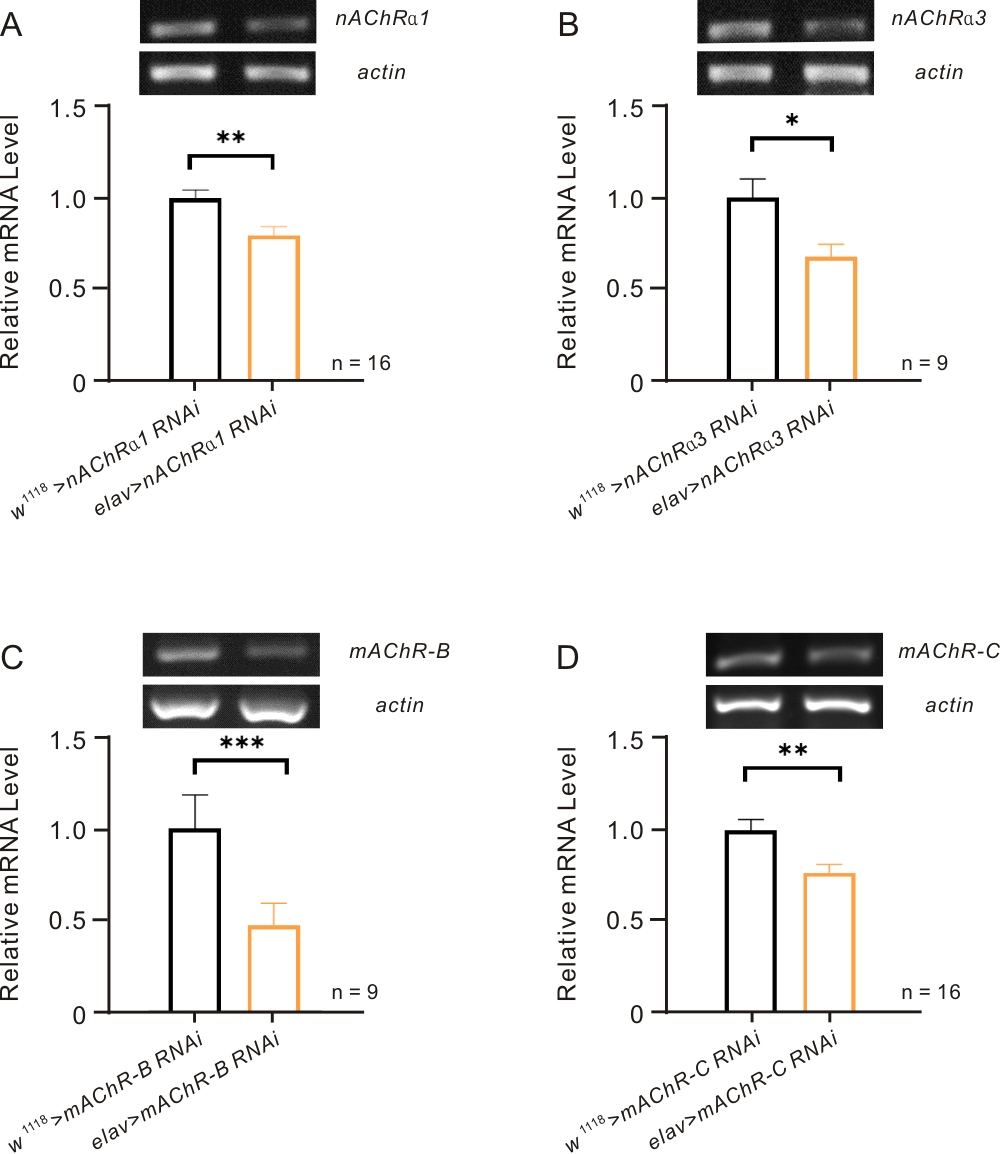

Supplement: Supplementary Figure 1 — The mRNA expression levels of different AChRs in Drosophila brains were suppressed using the RNAi technique. The RT-PCR results shown are expressed as the ratio of AChR to actin. The mRNA expression of nAChRα1 (A), nAChRα3 (B), mAChR-B (C), and mAChR-C (D) was significantly reduced in RNAi knockdown lines compared with compared with their corresponding control groups (*p < 0.05, **p < 0.01, and ***p < 0.001, t-test). [file Image_1.TIF]
